# Variation in Type 2 Diabetes-Related Phenotypes among Apolipoprotein E-Deficient Mouse Strains

**DOI:** 10.1371/journal.pone.0120935

**Published:** 2015-05-06

**Authors:** Shuiping Liu, Jing Li, Mei-Hua Chen, Zhenqi Liu, Weibin Shi

**Affiliations:** 1 Department of Radiology & Medical Imaging, University of Virginia, Charlottesville, Virginia, United States of America; 2 Department of Medicine, University of Virginia, Charlottesville, Virginia, United States of America; 3 Biochemistry & Molecular Genetics, University of Virginia, Charlottesville, Virginia, United States of America; University of Catanzaro Magna Graecia, ITALY

## Abstract

We recently have found that apolipoprotein E-deficient (Apoe^-/-^) mice with the C57BL/6 background develop type 2 diabetes when fed a Western diet for 12 weeks. In the present study we constructed multiple Apoe^-/-^ mouse strains to find diabetes-related phenotyptic variations that might be linked to atherosclerosis development. Evaluation of both early and advanced lesion formation in aortic root revealed that C57BL/6, SWR/J, and SM/J Apoe^-/-^ mice were susceptible to atherosclerosis and that C3H/HeJ and BALB/cJ Apoe^-/-^ mice were relatively resistant. On a chow diet, fasting plasma glucose varied among strains with C3H/HeJ having the highest (171.1 ± 9.7 mg/dl) and BALB/cJ the lowest level (104.0 ± 6.6 mg/dl). On a Western diet, fasting plasma glucose rose significantly in all strains, with C57BL/6, C3H/HeJ and SWR/J exceeding 250 mg/dl. BALB/cJ and C3H/HeJ were more tolerant to glucose loading than the other 3 strains. C57BL/6 was sensitive to insulin while other strains were not. Non-fasting blood glucose was significantly lower in C3H/HeJ and BALB/cJ than C57BL/6, SM/J, and SWR/J. Glucose loading induced the 1st and the 2nd phase of insulin secretion in BALB/cJ, but the 2nd phase was not observed in other strains. Morphological analysis showed that BALB/cJ had the largest islet area (1,421,493 ± 61,244 μm^2^) and C57BL/6 had the smallest one (747,635 ± 41,798 μm^2^). This study has demonstrated strain-specific variations in the metabolic and atherosclerotic phenotypes, thus laying the basis for future genetic characterization.

## Introduction

Atherosclerotic arterial disease is the leading cause of morbidity and mortality among individuals with type 2 diabetes mellitus (T2DM). The lifetime risk of a major cardiac event is 2 to 4 times higher in diabetic patients than in non-diabetic subjects [1]. Coronary heart disease, stroke, and peripheral arterial disease also occur at an earlier age compared to the general population [2]. The reasons for diabetes accelerated atherosclerosis are not completely understood, although hyperglycemia, dyslipidemia, and defective insulin signaling have been suggested to play a role [3]. Hyperglycemia induces a number of alterations at the level of vascular walls, specifically endothelial dysfunction, which accelerate the atherogenic process [4]. Diabetic patients often have dyslipidemia, which is a key factor in the development of atherosclerosis [5], and exhibit an atherogenic lipid profile, including an enrichment of triglycerides in the HDL core [6] and an increase in small-dense, triglyceride-rich LDL particles [7]. Loss of insulin signaling in endothelial cells accelerates atherosclerosis in Apolipoprotein E-deficient (Apoe^-/-^) mice [8], and insulin receptor deficiency in macrophages increases necrotic core size in advanced atherosclerotic lesions [9].

Apoe^–/-^ mice is a commonly used animal model of atherosclerosis, which develops all phases of atherosclerotic lesions, progressing from the early fatty streak stage to the advanced stage with a fibrous cap and necrotic lipid core [10]. Moreover, they display the typical features of dyslipidemia observed in humans, including elevations in LDL cholesterol and triglyceride levels and reductions in HDL cholesterol levels [11][12]. We have found that Apoe^–/–^ mice with the C57BL/6J (B6) genetic background develop type 2 diabetes and accelerated atherosclerosis when fed a Western diet, but they become resistant to both diseases when switched onto the BALB/cJ (BALB) background [13][14]. The variations among mouse strains in susceptibility to T2DM versus susceptibility to atherosclerosis provide an experimental means for finding intrinsic connections between the two diseases. Inbred mouse strains B6, C3H/HeJ (C3H), BALB, SWR/J (SWR), and SM/J (SM) have been shown to vary greatly in their susceptibility to atherosclerosis and plasma lipid levels when challenged with an atherogenic diet containing high fat, high cholesterol and cholate [15][16][17]. In this study, we transferred these strains onto the Apoe^–/-^ background to test the hypothesis that variations in the susceptibility to atherosclerosis could be attributable, at least partially, to variations in diabetes-related phenotypes.

## Results

### Atherosclerosis susceptibility

**Fig 1:**
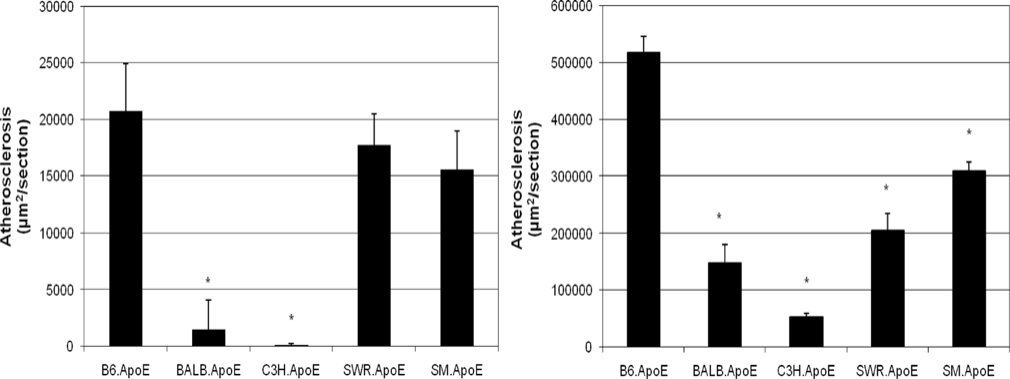
Atherosclerotic lesions in the aortic root of five Apoe^-/-^ mouse strains fed a chow or Western diet. Female mice were maintained on a regular chow diet after being weaned and euthanized at 12 weeks of age (Fig 1 left chart) or were started with a Western diet at 6 weeks of age and maintained on the diet for 12 weeks (Fig 1 right chart). Results are means ± SE of 6 to 18 mice. * *P* < 0.05 vs. B6 mice.

6 to 18 female mice from each of the 5 Apoe^-/-^ mouse strains were evaluated for lesion formation in the aortic root when fed the chow or Western diet. Atherosclerotic lesion sizes differed greatly among the 5 Apoe^-/-^ mouse strains with either diet. On the chow diet, B6, SWR, and SM Apoe^-/-^ mice formed significantly larger atherosclerotic lesions than C3H and BALB Apoe^-/-^ mice with *P* values in the range of 0.012 to 0.00013 (Fig 1 and Table A in S1 text). B6.Apoe^-/-^ mice had the largest aortic lesions, with an average lesion area of 20,733 ± 4,209 μm^2^/section (n = 16), followed by SWR.Apoe^-/-^ with a lesion area of 17,756 ± 2,707 μm^2^/section (n = 11) and SM.Apoe^-/-^ with an area of 15,575 ± 3,412 μm^2^/section (n = 8). In contrast, C3H.Apoe^-/-^ and BALB.Apoe^-/-^ mice developed small aortic lesions, with an area of 879 ± 311 (n = 6) and 1,510 ± 823 μm^2^/section (n = 10), respectively.

After being fed the Western diet for 12 weeks, B6.Apoe^-/-^ mice developed large aortic lesions, with an average lesion area of 518,648 ± 27,979 μm^2^/section (n = 18) (Fig 1 and Table A in S1 text). In contrast, C3H.Apoe^-/-^ mice had only a lesion area of 44,029 ± 5,221 μm^2^/section (n = 11). BALB.Apoe^-/-^ mice were also relatively resistant to atherosclerosis, with a lesion area of 148,667 ± 32,000 μm^2^/section (n = 9). SM.Apoe^-/-^ and SWR.Apoe^-/-^ mice were relatively susceptible to atherosclerosis, having lesion areas of 310,020 ± 15,083 (n = 10) and 206,202 ± 29,432 μm^2^/section (n = 15), respectively.

### Fasting plasma glucose levels

**Fig 2:**
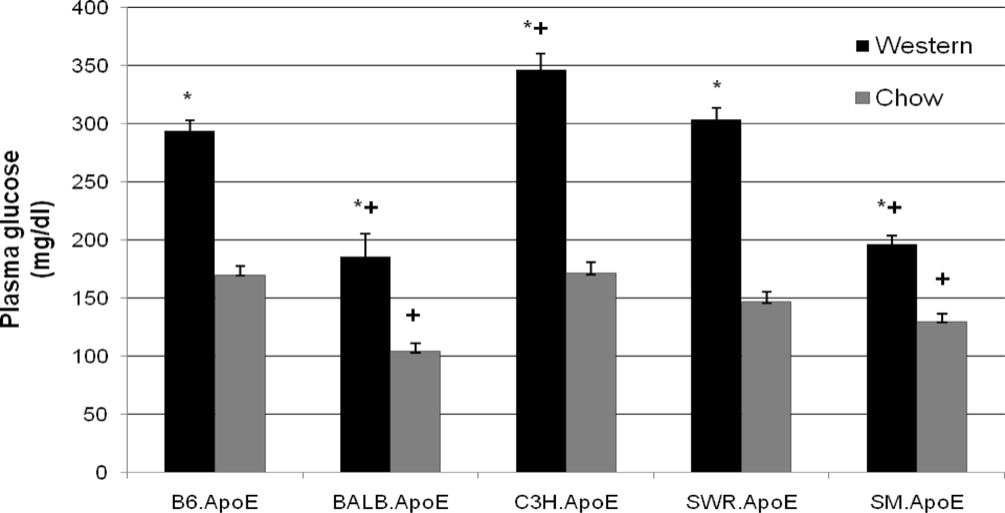
Fasting plasma glucose levels of five Apoe^-/-^ mouse strains fed a chow or Western diet. Blood samples were collected from overnight fasted mice. Results are means ± SE of 6 to 18 mice. * *P* < 0.05 vs. chow diet; ^+^ *P*< 0.05 vs. B6.Apoe^-/-^ mice.

On the chow diet, C3H.Apoe^-/-^ mice had the highest fasting plasma glucose level among the 5 strains, with an average of 171.1 ± 9.7 mg/dl (n = 6), and BALB.Apoe^-/-^ mice had the lowest level, with an average of 104.0 ± 6.6 mg/dl (n = 9) (Fig 2 and Table B in S1 text). The other 3 strains, including B6, SWR, and SM Apoe^-/-^ mice, had glucose levels falling in between, with an average of 170.1 ± 7.3 (n = 16), 146.4 ± 8.7 (n = 13), and 133.3 ± 7.2 mg/dl (n = 9), respectively. Compared to B6.Apoe^-/-^ mice, BALB and SM Apoe^-/-^ mice had significantly lower fasting glucose levels (*P*<0.0028). After 12 weeks on the Western diet, all 5 strains showed significant rises in fasting glucose levels (*P*<0.004). B6, C3H, and SWR Apoe^-/-^ mice had fasting glucose levels exceeding 250 mg/dl, with C3H having a level of 346.5 ± 14.4 mg/dl (n = 13), followed by SWR with 304.0 ± 9.9 mg/dl (n = 11) and B6 with a level of 294.0 ± 8.7 mg/dl (n = 17). BALB and SM Apoe^-/-^ mice had relatively low glucose levels, with values of 186.0 ± 19.7 mg/dl (n = 8) and 196.2 ± 7.9 mg/dl (n = 10), respectively. The difference between the high and the low glucose groups was statistically significant, with *P* values in the range of 0.0053 to 3.15 x 10^-8^.

### Glucose tolerance test (GTT) and insulin tolerance test (ITT)

**Fig 3:**
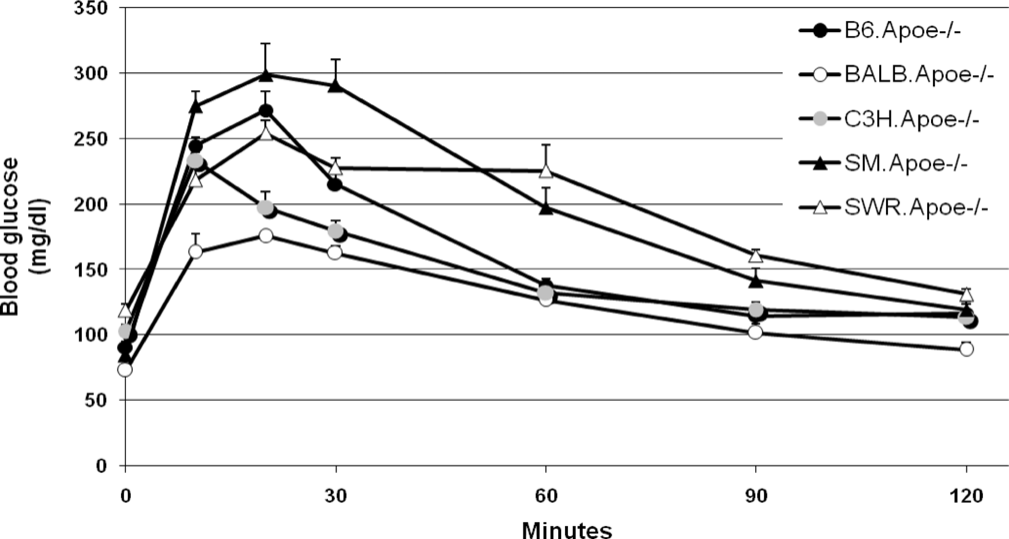
Glucose tolerance test (GTT) for five Apoe^-/-^ mouse strains fed a Western diet. For the test, overnight fasted mice were subject to an intraperitoneal injection of glucose (1 g/kg). Blood glucose concentrations were determined with a glucometer using blood taken from cut tail tips at the indicated time points. Values are means ± SE of 5 to 16 mice. When compared to B6 mice, all strains show statistical significance.

**Fig 4:**
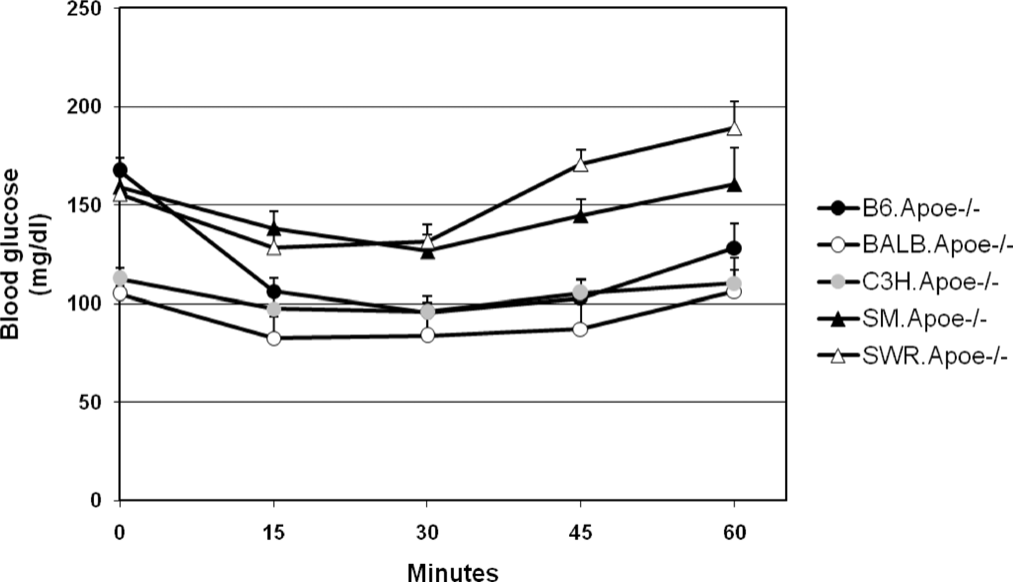
Insulin tolerance test (ITT) for five Apoe^-/-^ mouse strains fed a Western diet. Non-fasted mice were subject to an intraperitoneal injection of insulin (0.75 U/kg). Blood glucose concentrations were determined with a glucometer. Values are means ± SE of 5 to 16 mice. When compared to B6 mice, all strains show statistical significance.

GTT and ITT were performed on 5 to 16 mice per strain after 10 to 11 weeks of Western diet consumption. In response to intraperitoneally injected glucose, blood glucose levels quickly rose to the peak at the 10^th^ min for C3H or at the 20^th^ min for other 4 strains (Fig 3 and Table C in S1 text). Once reaching the peak, glucose levels started to fall for all strains, but the initial fall was faster for B6 and C3H relative to other strains. Compared to B6 mice, BALB and C3H mice had significantly lower glucose levels both at the peak and over the entire curve on the GTT (*P*<0.0067). On the contrary, SM and SWR mice displayed significant increases or a trend of significant increases in blood glucose at the peak and over the entire curve on GTT (P values: 0.0497 to 0.173). In response to insulin, B6.Apoe^-/-^ mice showed a deeper and longer-lasting fall in blood glucose levels (Fig 4 and Table D in S1 text). The other 4 strains also showed a fall in blood glucose, but the magnitude of falling was much smaller. Interestingly, the basal (at 0 min) non-fasting blood glucose levels were significantly lower in BALB (104.8 ± 4.2 mg/dl) and C3H (112.4 ± 5.5 mg/dl) than the levels of B6 (167.3 ± 6.8 mg/dl), SM (165.2 ± 5.2 mg/dl) and SWR (155.5 ± 3.6 mg/dl) (*P* values: 1.7 x 10^-5^ to 1.4 x 10^-7^).

### Glucose-stimulated insulin secretion

**Fig 5:**
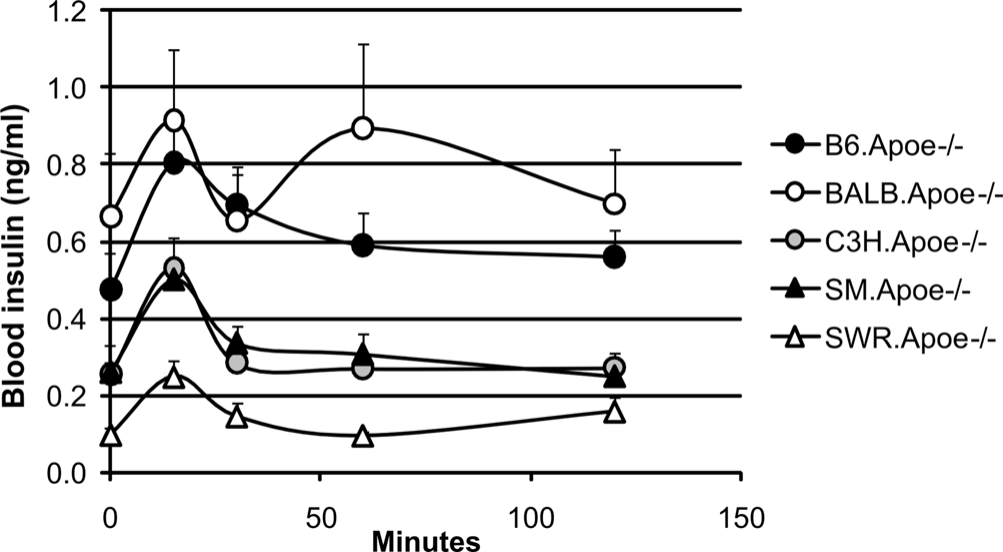
Glucose-stimulated insulin secretion in five Apoe^-/-^ mouse strains fed a Western diet. Mice were fasted overnight and then injected intraperitoneally with glucose (1 g/kg). Blood was collected from cut tail tips. Values are means ± SE of 5 to 12 mice for each strain. When compared to B6 mice, all strains show statistical significance.

The test was performed on 4 to 12 mice fed the Western diet for each strain. In response to glucose, BALB.Apoe^-/-^ mice exhibited both the first phase and the second phase of insulin secretion while the second phase of insulin secretion was not observed in other 4 strains (Fig 5 and Table E in S1 text). For BALB, the first peak of insulin secretion appeared at the 15^th^ min after glucose injection and the second peak occurred at the 60^th^ min; for the other 4 strains, the peak of insulin secretion also appeared at the 15^th^ min, but by the 60^th^ min insulin levels fell to the pre-stimulated level. In addition, these 5 strains differed in their basal insulin levels, in the order of BALB (0.664 ± 0.166 ng/ml; n = 10) > B6 (0.472 ± 0.096; n = 11) > SM (0.258 ± 0.035; n = 10) > C3H (0.253 ± 0.030; n = 12) > SWR (0.095 ± 0.020 ng/ml; n = 4).

### Islet mass

**Fig 6:**
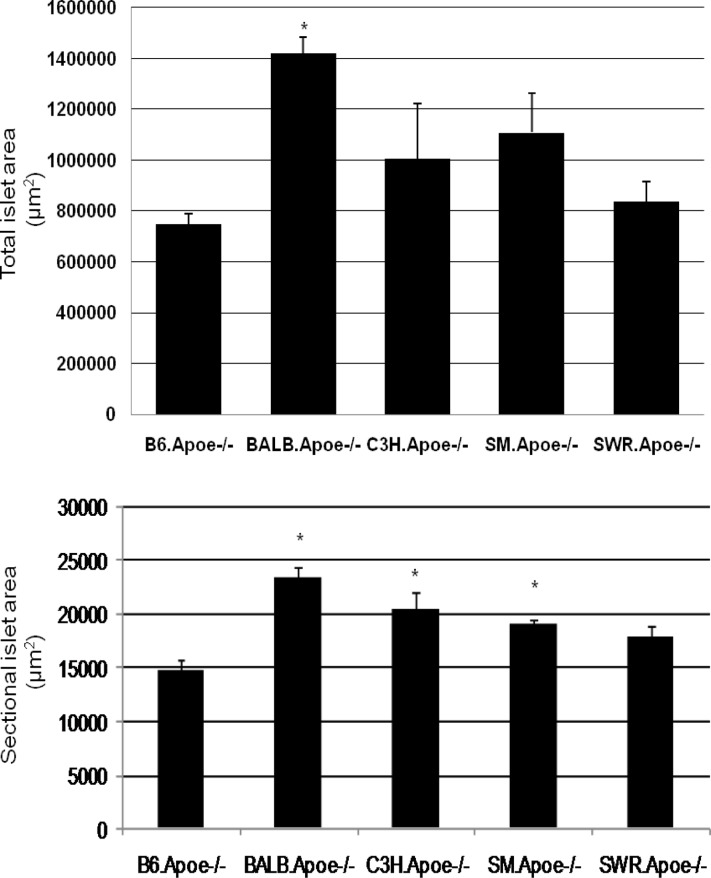
Total (upper panel) and sectional (lower panel) pancreatic islet surface areas of five Apoe^-/-^ mouse strains fed a Western diet. Cryosections of the pancreas were stained with haematoxylin and eosin. The surface area of all pancreatic islets from sections spaced approximately at 500 μm was measured for each mouse. Values are means ± SE of 3 to 11 mice. * *P* < 0.05 vs. B6 mice.

BALB.Apoe^-/-^ mice had the largest total cross-sectional area of islets (1,421,493 ± 61,244 μm^2^; n = 7), which was approximately 2-fold as large as that of B6.Apoe^-/-^ mice (747,635 ± 41,798 μm^2^; n = 11) (Fig 6 and Table F in S1 text). The difference was highly significant (*P* = 1.28x10^-6^). The other 3 strains of mice were also different in their islet cross-sectional areas, with the order of SM (1,108,408 ± 155,777 μm^2^; n = 3) > C3H (1,005,752 ± 216,925 μm^2^; n = 5) > SWR (837,007 ± 79,532 μm^2^; n = 5). BALB and B6 mice also had the largest and the smallest cross-sectional area (23,443 ± 987 and 14,710 ± 1,084 μm^2^; *P* = 2.85 x 10^-5^), respectively. The cross-sectional islet areas of C3H and SM mice were 20,461 ± 1,594 and 19,174 ± 343 μm^2^, respectively, significantly larger than the area of B6 mice (*P*< 0.019). SWR mice also had a larger cross-sectional islet area (17,900 ± 1,019 μm^2^) than B6 mice, but the difference did not reach statistical significance (*P* = 0.058).

### Body weight

**Fig 7:**
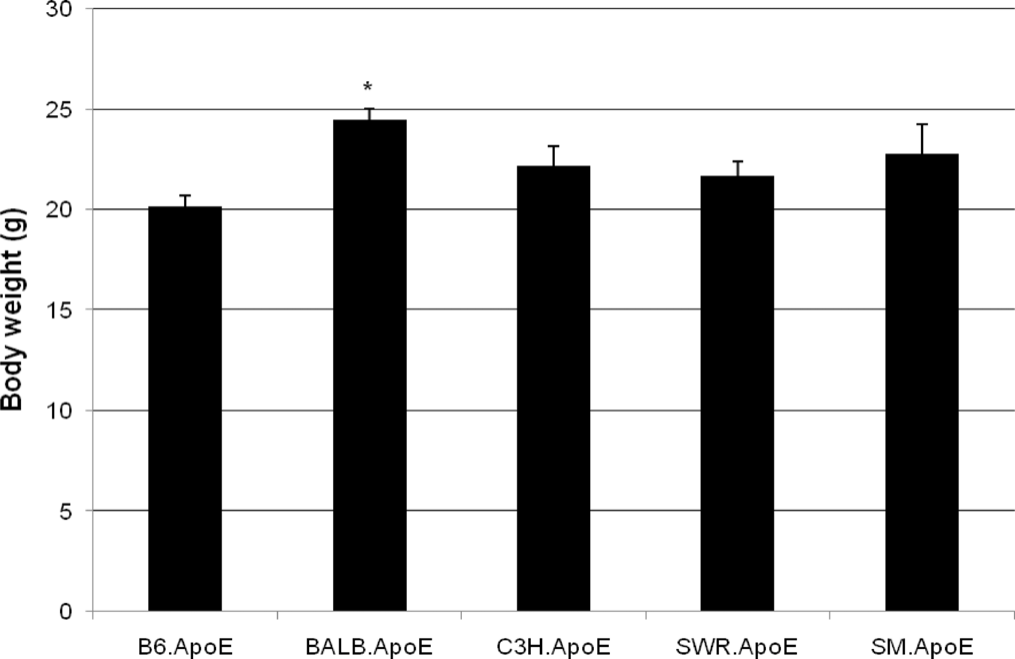
Body weight (g) of five Apoe^-/-^ mouse strains fed a Western diet. Results are means ± SE of 6 to 18 mice. * *P* < 0.05 vs. B6 mice.

BALB.Apoe^-/-^ mice had a body weight of 24.5 ± 0.6 (n = 9) and, significantly larger than that of B6.Apoe^-/-^ mice (20.2 ± 0.5 g; n = 18; *P* < 0.012) (Fig 7 and Table G in S1 text). SM, C3H, and SWR Apoe^-/-^ mice had body weight of 22.8 ± 1.4 (n = 10), 22.2 ± 1.0 (n = 11), and 21.7 ± 0.7 (n = 15) g, larger than the weight of B6.Apoe^-/-^ mice, but the difference was not statistically significant (*P* values in the range of 0.094 and 0.11).

### Plasma lipid levels

**Fig 8:**
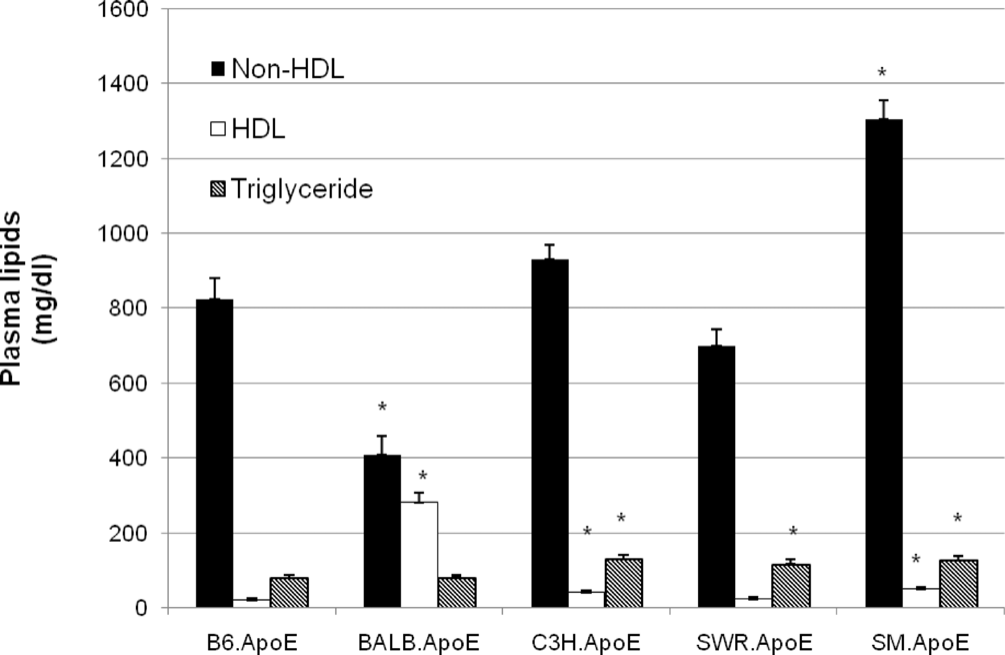
Plasma non-HDL and HDL cholesterol and triglyceride levels of five Apoe^-/-^ mouse strains fed a Western diet. Blood was obtained after overnight fastening. Values are mean ± SE for 6 to 18 mice. *P<0.05 vs B6 mice.

Plasma non-HDL (LDL and VLDL) cholesterol, HDL cholesterol and triglyceride levels were measured for 6 to 18 mice per strain fed the Western diet (Fig 8 and Table H in S1 text). SM.Apoe^-/-^ mice had the highest non-HDL cholesterol level among the 5 strains, with an average of 1,304.8 ± 48.8 mg/dl (n = 10), and BALB.Apoe^-/-^ mice had the lowest non-HDL level, with an average of 410.1 ± 43.0 mg/dl (n = 12), significantly different from that of B6.Apoe^-/-^ mice (824.7 ± 56.1 mg/dl; n = 18; *P* = 1.19E-6 and 3.6E-6 respectively). C3H.Apoe^-/-^ mice had an average non-HDL level of 930.2 ± 39.8 mg/dl (n = 13) and SWR.Apoe^-/-^ mice had an non-HDL level of 699.7 ± 43.1 mg/dl (n = 11), both of which were not significantly different from that of B6.Apoe^-/-^ mice (*P* = 0.137 and 0.089, respectively). HDL cholesterol levels also varied considerably among the 5 strains, with BALB having a level of 283.0 ± 22.2 mg/dl and with B6 having a level of 21.9 ± 4.1 mg/dl. SWR.Apoe^-/-^ mice also had a HDL level of 23.0 ± 4.6 mg/dl, comparable to the level of B6 (*P* = 0.20) but lower than the levels of 50.0 ± 5.6 and 42.8 ± 2.9 mg/dl in SM and C3H mice (*P*<0.0012). The triglyceride levels also varied among the 5 strains with C3H and SM mice having significantly higher levels (130.8 ± 10.4 and 127.1 ± 11.4 mg/dl, respectively) than B6 (80.3 ± 8.1 mg/dl), SWR (117.1 ± 12.1 mg/dl) and BALB (81.9 ± 5.7 mg/dl) (*P*<0.05).

## Discussion

In this study, we created multiple Apoe^-/-^ mouse strains to find phenotypic interaction between atherosclerosis and type 2 diabetes. B6, SM, and SWR Apoe^-/-^ mice were found to be susceptible to atherosclerosis, intolerant to glucose loading and have impairments in non-fasting blood glucose on the Western diet. On the contrary, C3H and BALB Apoe^-/-^ mice were found to be resistant to atherosclerosis, tolerant to glucose loading and have low non-fasting blood glucose. However, no distinctive patterns were found between the atherosclerosis-susceptible strains and the resistant strains in fasting glucose, insulin resistance, body weight, islet mass, and fasting lipids.

The susceptibility of 5 Apoe^-/-^ mouse strains to atherosclerosis was determined by assessing plaque formation in the aortic root. At 12 weeks of age on a chow diet, B6, SM, and SWR Apoe^-/-^ mice developed much larger lesions than C3H and BALB Apoe^-/-^ mice. This finding is in agreement with the previous observations that mouse strains B6, SM and SWR are susceptible to fatty streak lesion formation and that C3H and BALB are resistant [17][16]. In the previous studies, mice were fed an atherogenic diet containing high fat/cholesterol and cholate for 14 to 18 weeks, and with this treatment they developed fatty streak or early stage lesions. Apoe^-/-^ mice of 12 weeks of age have been shown to develop fatty streak lesions on a chow diet [10]. After 12 weeks on the Western diet, Apoe^-/-^ mice should have developed advanced lesions [10]. The relative susceptibility of 5 Apoe^-/-^ strains to atherosclerosis remained largely unchanged with the Western diet, although the aortic lesion area of B6 mice had doubled the area of SWR or SM mice.

One major finding of the present study was the distinct patterns displayed between atherosclerosis-susceptible strains and resistant strains in non-fasting glucose levels and glucose tolerance on the Western diet. The atherosclerosis-resistant strains C3H and BALB had significantly lower non-fasting blood glucose and displayed greater glucose tolerance than the susceptible B6, SM and SWR strains. Epidemiological studies have revealed a close link between postprandial hyperglycemia and cardiovascular mortality in different ethnic groups [18][19][20]. As atherosclerosis susceptibility was defined based on plaque formation in this study, these findings may suggest that the increased mortality in individuals with postprandial hyperglycemia is probably due to accelerated plaque formation.

In this study, we found no distinction between atherosclerosis-prone and resistant strains in fasting glucose. Rather, atherosclerosis-resistant C3H mice had as high a fasting glucose level as that of atherosclerosis-prone B6 and SWR mice, and atherosclerosis-prone SM mice had a fasting plasma glucose level that was almost as low as that of atherosclerosis-resistant BALB mice. In humans, fasting glucose levels are often found to be less strongly associated with cardiovascular risks than postprandial fasting glucose values [21].

A paradoxical finding is that C3H mice had an extremely high fasting glucose level but a low non-fasting glucose level on the Western diet. In contrast, BALB mice had low fasting and non-fasting glucose levels on the same diet. Both strains also exhibited high insulin resistance, but C3H mice showed low basal and stimulated insulin secretion. These findings suggest that low insulin secretion was probably responsible for fasting hyperglycemia observed in C3H mice. Differential effects of defective insulin resistance versus secretion on fasting and postprandial glucose have been investigated on identical twins with type 2 diabetes [22], hemipancreatectomized individuals [23], and insulin resistant individuals [24], and the results have collectively shown that impaired insulin secretion preferentially affects fasting glucose and primary insulin resistance preferentially affects postprandial glucose. Rates of endogenous glucose production have been shown to be proportional to fasting plasma glucose concentrations [25]. Thus, it was also likely that the elevated level of fasting glucose in C3H mice could be partially due to an increase in endogenous glucose production under the fasting condition.

We have found that the Apoe^-/-^ mouse strains except for BALB had impairments in insulin secretion on the Western diet. Insulin secretion following glucose loading is usually biphasic, consisting of a temporary initial peak of insulin release, followed by a prolonged second phase. The first phase insulin secretion was quite comparable among the strains, but the second phase insulin response was only observed in BALB mice. The basal insulin level also varied greatly among the mouse strains. BALB/c mice had a relatively large islet mass, which may contribute to the high level of insulin secretion, the strong tolerance to glucose and the high resistance to diabetes compared to other strains.

Despite insulin resistance, BALB mice developed no diabetes due to normal β-cell function. In contrast, B6 mice had no impairment in insulin sensitivity but developed diabetes. These results suggest that defective β cell function is essential to the development of diabetes. The recent genome-wide association studies have identified much more new loci that are implicated in β-cell development and function than those implicated in insulin action, highlighting insulin secretion in the development of type 2 diabetes in humans as well [26].

In mice fasting plasma glucose levels exceeding 250 mg/dl is considered diabetic [27]. Based on this criterion, B6, C3H, and SWR mice had developed diabetes on the Western diet, but BALB and SM had not. We found that none of the mouse strains developed obesity on the diet. This finding is in agreement with previous observations that Apoe deficiency prevents the development of obesity in B6 mice and genetically obese Ay mice on a high fat diet [28][29]. Although obesity is associated with 60–80% of type 2 diabetic cases in the Europeans and Africans, it is only associated with 30% of cases in the Chinese and Japanese [30]. Despite the absence of obesity, all strains, except for B6, developed insulin resistance on the Western diet. Obviously, insulin resistance alone could not explain the variation observed among the strains in susceptibility to diet-induced diabetes. In a recent study of 5 wild-type mouse strains, including B6 and BALB, Montgomery et al [31] observed the presence of significant insulin resistance, which was partially attributable to a significant gain of body fat on a high fat diet. That study was carried out with male mice and there might be sex differences in development of obesity and insulin resistance.

On the Western diet, BALB mice had much higher HDL levels than the other 4 Apoe^-/-^ strains. C3H and SM mice also had higher HDL levels than B6 and SWR mice. Besides its anti-atherogenic properties, HDL has anti-diabetic effects, including promotion of the uptake of glucose by skeletal muscle [32] and stimulation of the synthesis and secretion of insulin from pancreatic β cells [33]. Thus, it was likely that a higher HDL level in atherosclerosis-resistant strains resulted in an increased level of HDL-mediated protection and a reduced risk of developing diabetes. SM and C3H mice had higher total cholesterol levels than other 3 strains. C3H also exhibited a slightly but statistically higher triglyceride level than B6 mice and BALB mice displayed a lower triglyceride level than B6 mice. Nevertheless, the differences in non-HDL cholesterol and triglyceride among the 5 mouse strains could not explain the variations in their susceptibility to atherosclerosis or diabetes.

The Western diet used in this study contains a large fraction of sucrose (35% by weight) and fat (21%). This type of diet induces steatohepatitis that is dependent on fructokinase in mice [34]. The *Khk* gene, encoding fructokinase, contains no polymorphisms in coding or upstream regulatory regions that can change the function or expression of the enzyme among the 5 strains tested. Though there are a few SNPs in the intron regions of *Khk*, these SNPs do not segregate with variations in atherosclerosis or glucose levels among the strains.

In the present study, we found that Apoe deficiency significantly sensitized high-fat diet-induced hyperglycemia in the mouse strains tested. In contrast, high-fat diet only induces little or moderate rises in blood glucose of wild-type mouse strains [31][35]. The Apoe gene in the mouse is orthologous to APOE3 in humans, who also have APOE2 and APOE4 isoforms. Recent studies have shown that APOE genotypes are associated with risk of T2DM and complications in humans. For example, the ApoE2 and ApoE4 alleles are associated with risk of T2DM and/or diabetic nephropathy in Han Chinese and Thai [36][37], although no associations were found in type 2 diabetic Turkish patients [38].

In summary, we have provided the first experiment evidence supporting the intrinsic connections between atherosclerosis and type 2 diabetes using the Apoe^-/-^ mouse model. The clinical observation that nearly 80% of Asian patients with coronary artery disease had abnormal glucose tolerance [39] supports that this is the case for humans as well. Importantly, we have demonstrated that elevated non-fasting blood glucose and glucose intolerance are more closely linked to atherosclerosis susceptibility relative to insulin resistance and fasting glucose in the Apoe^-/-^ mouse stains. Furthermore, the phenotypic interaction between atherosclerosis and metabolism observed in the inbred strains has laid the basis for positional cloning torward identification of responsible genes as pursued in our previous work [40][41][42].

## Materials and Methods

### Ethics statement

All procedures were carried out in accordance with current National Institutes of Health guidelines and approved by the University of Virginia Animal Care and Use Committee (Assurance #A3245-01, Animal Protocol #3109).

### Mice

All Apoe^-/-^ strains were backcrossed 9 generations or more to their respective background strains. B6.Apoe^-/-^ mice were purchased from the Jackson Laboratory, Bar Harbor, ME. C3H/HeJ (C3H), BALB, SM/J (SM) and SWR/J (SWR) Apoe^-/-^ mice were created in our laboratory using the classical congenic breeding protocol [43]. Briefly, B6 mice carrying the mutant Apoe^-/-^ gene were crossed with C3H mice, and the resultant F_1_ heterozygotes were then backcrossed to C3H mice. The N_2_ progeny carrying the heterozygous Apoe^+/-^ gene were selected and backcrossed to C3H mice again. This procedure was repeated until the N_10_ generation was generated. Heterozygous Apoe^+/-^ mice were intercrossed to generate homozygous C3H.Apoe^-/-^ mice. The same breeding scheme was used to generate BALB.Apoe^-/-^, SM.Apoe^-/-^, and SWR.Apoe^-/-^ mice. At 6 weeks of age, mice continued with a chow diet containing 19% protein, 5% fat, 5% crude fiber, and 3.1 kcal/g in energy density with 25% of the calories from protein, 17% from fat, and 58% from carbohydrate (Teklad LM-485, Harlan Laboratories; http://www.harlan.com) for additional 6 weeks, or were switched onto a Western diet containing 21% fat, 0.15% cholesterol, 34.1% sucrose, 19.5% casein, 15% starch, and 4.5 kcal/g in energy density with 15% of the calories from protein, 42% from fat, and 43% from carbohydrate (TD88137, Harlan Laboratories;) and maintained on the diet for 12 weeks. The mice were housed in a 12-h light/12-h dark cycle pathogen-free facility at the University of Virginia.

### Measurements of plasma glucose and lipids

Mice were fasted overnight before blood was collected from the retro-orbital venous plexus by inserting a micro-hematocrit capillary with the animals under isoflurane anesthesia. Ethylenediaminetetraacetic acid (EDTA) was used as an anti-coagulant. Collected blood samples were centrifuged within 1 h at 12,000 g for 5 min at 4°C, and the resulting plasma was stored at -80°C before assay. Plasma glucose was measured with a Sigma glucose (HK) assay kit [44], plasma total cholesterol, HDL cholesterol and triglyceride were measured with Thermo DMA cholesterol and triglyceride kits, as we reported [14]. Non-HDL cholesterol was calculated as the difference between total and HDL cholesterol levels.

### Atherosclerotic lesion analysis

Atherosclerotic lesions in the aortic root were measured as we reported [45,42]. Briefly, upon termination of the experiments, the vasculature of mice was perfused with 4% paraformaldehyde (PFA) for approximately 5 min through the heart. The aortic root and adjacent heart were excised, embedded in OCT compound and sectioned in 10-μm thickness on a cryostat. In the region from the appearance to the disappearance of the aortic valves, every other section was collected. In all other regions, every fifth section was collected. Sections were stained with oil red O and hematoxylin and counterstained with fast green. Atherosclerotic lesions were quantified by light microscopy. The lesion areas of five sections with the largest readings were averaged for each mouse, and this average was used for statistical analysis.

### Glucose tolerance test (GTT) and insulin tolerance test (ITT)

The tests were performed after 10 or 11 weeks of Western diet consumption. These tests were performed by following the UVA Animal Characterization Core established protocols as described by others [46][47]. The same or similar ITT protocol has been used by other investigators [31][48][49]. For GTT, mice were fasted overnight and then subject to an intraperitoneal injection of glucose (1g/kg). Whole blood glucose was measured with a glucometer using blood squeezed from cut tail tips immediately before and at 10, 20, 30, 60, 90, and 120 min after the injection of glucose. ITT was performed on non-fasted mice starting between 1 to 2 PMby an intraperitoneal injection of insulin (0.75 U/kg). Blood glucose was measured immediately before and at 15, 30, 45, and 60 min after insulin injection.

### Glucose-stimulated insulin secretion

Evaluation of insulin secretion was performed as we described [13]. Briefly, overnight fasted mice were injected intraperitoneally with glucose (1 g/kg). Blood samples were collected from cut tail tips immediately before and at 15, 30, 60, and 120 min after glucose injection and dissolved in a sample buffer provided in an ELISA kit from Crystal Chem INC. Blood cells were removed by centrifugation before insulin concentrations in the samples were measured with the ELISA kit, capable of detecting insulin as low as 0.1 ng/ml.

### Evaluation of islet mass

Pancreatic islet mass was evaluated with a histological method as we described [13]. Briefly, the whole pancreas was harvested, fixed in 4% paraformaldehyde (PFA) for over 24 h, then soaked overnight in 30% sucrose, and sectioned on a cryostat. Cryosections in 8-μm thickness were collected every 3 sections throughout the pancreas, and 4 sections were mounted on one slide. Every 5th slide was stained with haematoxylin and eosin (H&E), and the cross sectional areas of all islets in proximal and distal sections on each slide were counted. The average cross sectional areas of islets on each stained slide were added up for each mouse and this sum was used for statistical analysis.

### Statistical analysis

Values were expressed as means ± SE, with *n* indicating the number of mice. The distribution of data was examined for normality with SPSS software. ANOVA or Student's t test were used for determining statistical significance between groups when data were roughly normally distributed or at least symmetric. Differences were considered statistically significant at *P* < 0.05.

## Supporting Information

S1 Text *Supporting Tables*: Characterization of 5 Apoe-/- mouse strains in atherosclerosis and type 2 diabetes related phenotypes. (XLSX)
